# Clinical Evaluation of Bond Failures and Survival of Mandibular Canine-to-canine Bonded Retainers during a 12-year Time Span

**DOI:** 10.5005/jp-journals-10005-1460

**Published:** 2017-02-27

**Authors:** Marcello Maddalone, Elisa Rota, Luca Mirabelli, Pier M Venino, Gianluca Porcaro

**Affiliations:** 1 Chief, Department of Medicine and Surgery, University of Milano-Bicocca, Milan, Italy; 2 House Officer, Department of Medicine and Surgery, University of Milano-Bicocca, Milan, Italy; 3 House Officer, Department of Medicine and Surgery, University of Milano-Bicocca, Milan, Italy; 4 House Officer, Department of Medicine and Surgery, University of Milano-Bicocca, Milan, Italy; 5 House Officer, Department of Medicine and Surgery, University of Milano-Bicocca, Milan, Italy

**Keywords:** Bonded lingual splint, Orthodontic relapse, Retention.

## Abstract

**Aim:**

The aim of this study was to assess the effectiveness of the 3-3 mandibular lingual stainless steel retainer to prevent a relapse of orthodontic treatment during the 12-year time span of the survey.

**Materials and methods:**

Fifty patients with canine-to-canine bonded retainers (placed at least 10 years earlier) were recalled. All patients had been followed up annually during this period. Patients were screened for stability of the retainer and for the condition of hard and soft oral tissues.

**Results:**

None of the patients reported a complete loss of the retainer; 14 patients reported single element partial losses and 13 reported multiple losses. Most partial failures were not perceived by patients, but noted by the orthodontist during the control visit. There was no notable variation of the gingival index occurring in these patients. In two cases patients had caries in the six teeth bonded with the retainer, but never on the lingual side; only in three teeth areas of decalcification in the proximity of bonded sites were reported. All patients showed good compliance with this kind of retention.

**Conclusion:**

The composite adhesive technique allowed a reliable positioning system for directly bonded retainers and did not influence the occurrence of carious lesions or demin-eralized spots on fixed teeth. Full teeth fixation offered the possibility of stabilizing the irregularity index highlighted in various studies without increasing any side effects on gums and hard tissues.

**How to cite this article:** Maddalone M, Rota E, Mirabelli L, Venino PM, Porcaro G. Clinical Evaluation of Bond Failures and Survival of Mandibular Canine-to-canine Bonded Retainers during a 12-year Time Span. Int J Clin Pediatr Dent 2017;10(4):330-334.

## INTRODUCTION

Throughout the past century and recent years, orthodontists have developed different methods to ensure the stability of teeth position achieved during treatments. Removable and fixed appliances were used to stabilize the alignment of mandibular incisors after active treatment in this particular area.

Edward Angle^1^ introduced the concept that occlusal forces against teeth aligned in a proper occlusion will tend to keep them in that position. Tweed demonstrated that a better stability could be gained with premolar extractions, retreating patients with relapse after fixed appliance treatment. Tweed^2^ also suggested that one of the major determinants in generating relapse was arch width expansion.

Edwards^3^ described the surgical technique to prevent rotational relapse, which has become known as circumferential supracrestal fiberotomy. This followed Reitan’s^4^ observations, which demonstrated that gingival elastic fibers contribute to relapse after correction of rotations. Andrews,^5^ more recently, pointed out the importance of musculature balance after treatment, and the need for special attention for cases in which abnormal environmental or hereditary factors are present. Nevertheless, other authors, including Edwards^3^ and Rinchuse et al,^6^ found that the effect of circumferential supracrestal fiberotomy was not complete in reducing tooth movement after therapy ended, especially with labiolingual relapses. In fact, these types of movement were not as stable in the maxilla as in the mandible and they were not stable over time.

Other factors affecting long-term stability were cited in the past as: The interincisal angle,^7^ intercanine width,^7,8^ overcorrection of rotations,^9^ and posttreatment growth changes.^10,11^

Various authors have shown that in untreated normal occlusions, arch length decreases and, in particular, mandibular incisor crowding increases throughout life.^12^ The changes, which are often accepted by the clinician as part of the normal maturational process, are considered as a relapse by patients and relatives.^13^ Therefore, at the end of this debate, we can understand that, as Booth et al^14^ state, “most orthodontists believe that stable treatment is a myth.”

In effect, stable treatments exit where occlusion can guarantee that, but in the lower anterior region, where it is impossible, our created artificial alignment is affected by the law of time passing, as with facelifts.

The final objective of an orthodontic treatment is to obtain a valid occlusal function, but this should also coincide with current esthetic demands. This justifies the use of fixed retainers at the end of a fixed appliance treatment to avoid changes that, with time, can alter the esthetic balance achieved previously. These systems are required particularly in the years following the end of treatment to counteract the force of the elastic fibers of the periodontium and to allow an adequate remodeling of the alveolar bone.^7,15-17^

Reliability of fixed retention appliances is crucial to obtain long-term stability of orthodontic results.^18^ In fact, the etiological factors of malocclusion should be determined at the time of the initial diagnosis and they should also be controlled during treatment and retention to prevent relapse.

Bonded retainers can be of different types: Bonded only to canine teeth, or to all six anterior mandibular teeth; made of different sections of wire; and made of different kinds of wire, multistranded or single. Different points of view generate different choices: For example, according to some authors, canine-to-canine only bonded retainers offer better cleaning possibilities.^6,10^

The results of a recent clinical study showed no statistically significant difference in failure rates between multistranded wire retainers and glass fiber-reinforced resin composite retainers over a 1-year follow-up period.^19^

The purpose of this study was to evaluate the retention stability of a multistranded round wire fixed to all six anterior mandibular teeth and the effect on periodontal tissues and enamel surfaces on a follow-up at 12 years.

## MATERIALS AND METHODS

For the study, we selected 50 patients (from the dental clinic of our Department of Orthodontics) who had orthodontic therapy with the use of a fixed appliance at least 12 years earlier. As retention device, all patients received a fixed splint made with a twisted multistranded stainless steel wire, passively adapted to the lingual side of the mandibular incisors and canines. Splints were made to be positioned near the cingulum of teeth and were fixed to all teeth to limit the possibility of rotational relapse and to decrease the possibility of deformation to the teeth-splint unit.

To reduce the possibility of irritation to periodontal tissues and to enable easy daily cleaning procedures, the composite used to fix the splint was applied just around the wires and at least 3 mm far from the gum, using a direct technique. Before the positioning of the splints, all dental surfaces were polished with pumice and rotor brushes.

Patients recruited for the study were contacted during the annual routine check visit. During the appointment, all surfaces were cleaned and carefully inspected for detection of bond failures. Bond failures were registered and immediately solved, if possible. When movements had occurred, corrections were made and, as soon as possible, the wire was resecured to the teeth.

A gingival index score was obtained for complete dentition using the scoring method according to Loe and Silness, with scores of: 0 for normal gingiva and absence of inflammation; 1 for mild inflammation, slight change of color, slight edema, no bleeding on probing; 2 for moderate inflammation, bleeding on probing; and 3 for severe inflammation, ulceration, tendency to spontaneous bleeding.

According to Booth et al,^14^ to generate a score, a probe was passed with minimal pressure from interproximal contact through the gingival sulcus to the next interproxi-mal contact, on both the lingual and the facial aspects of each tooth from first molar to first molar. A separate score was recorded for the facial and the lingual aspects of each tooth and these scores were averaged to generate a score for the anterior (canine to canine) and right and left posterior regions of both arches for each patient. Patients were also asked how many times they had experienced partial or complete loss of the retainer or of the composite. These data were cross-checked with the data originating from clinical records.

Similarly, the occurrence of carious lesions or white spots and decalcification areas was recorded through photographic records at individual appointments and at the end of active treatment.

Subjective compliance with the retainer used was also collected using a scale, where 10 means very good compliance, satisfaction with results, and no disturbances during the 10-year time span and 0 means no tolerance for the splint.

## RESULTS

From the pool of 50 patients who had completed orthodontic therapy with the use of a fixed appliance at least 12 years earlier, 46 (92%) still had the bonded splint in place at the time of the check-up. The other 4 had removed the splint for different reasons: 1 because of irritation to the surface of the tongue, and the other 3 for personal reasons.

**Graph 1:**
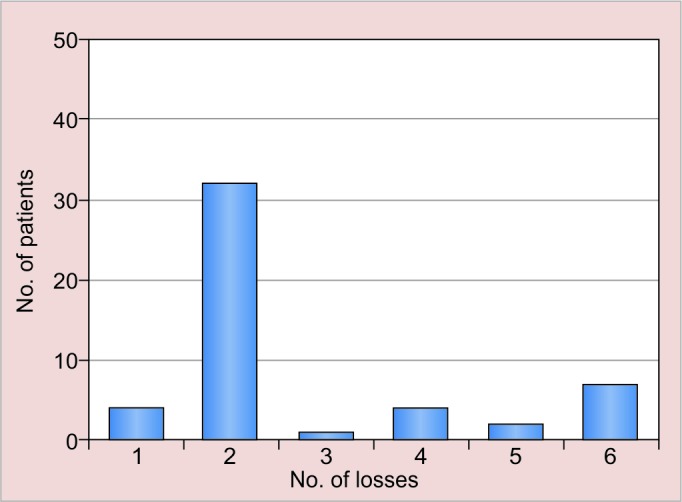
(1) Patients who wanted to remove the splint—8%; (2) no loss—64%; (3) 1 loss—2%; (4) 2 losses—8%; (5) 3 losses—4%; (6) 4 losses or more—14%—during the 12-year time span

Of the 46 patients who had the retainer in place at the check-up, 32 reported no loss during the 12-year time span, 14 (28%) had partial losses, whether or not associated to dental displacement. No one reported total loss of the retainer. Of these 14 patients, 13 reported more than 1 partial loss of adhesion in the 12-year time span considered: 7 reported 4 or more losses, 2 reported 3 losses, and 4 reported 2 losses (Graph 1).

None of the patients examined for this article showed alterations of the gingival index in the area where the splint was applied.

Of the 50 patients taken into consideration, only two showed carious lesions in the lower incisor area, and both were in the group of 46 that continued to wear the splint for the 12-year time span. In the same period, 18 others showed carious lesions in the posterior segment, especially in interproximal locations.

The two patients with lesions in the anterior region contemporarily had lesions in the posterior segments. Nevertheless, the two lesions were not in the lingual facets but in the vestibular one, and they were never in direct contact with wire or composite. Areas of decalcification were observed in three patients. In all of these cases, the areas of decalcification were in direct contact with the composite fixation of splints.

All patients who had kept the retainer in place for 12 years demonstrated good compliance with this method of retention, with an average score of 9.434 on a scale from 0 to 10.

## DISCUSSION

The results of this study are consistent with the thesis that supports the usefulness of totally bonded retainers in reducing the frequency of complete loss of the retainers. As Renkema et al^20^ state, “Because the stability of alignment was negatively affected by failures of a bonded retainer, it is important to stress the value of the periodic maintenance of retainers bonded to the canines.” In a review of the same article, Rinchuse et al^6^ noted that, “The current gold standard for mandibular canine to canine fixed retention usually includes bonding a wire to each of the 6 anterior teeth.”

On the contrary, the reported lack of total loss of the retainer during the period of observation reflects the close control of the bonded devices. This depends on the fact that all those followed were patients of a single clinician, who were regularly seen for hygiene, recall at least once a year, when the hygienist inspected the appliance and reported initial composite fractures or losses. This indicates that regular inspections are important to guarantee appropriate maintenance of these devices.

Furthermore, as Årtun^16^ stated, a retainer could have a positive effect on hygiene: “The presence of a retainer wire, with occasional accumulation of plaque and calculus, does not seem to prevent satisfactory hygiene along the gingival margin. In this regard, the patient’s own attitude and motivation, possibly acquired under the influence of the orthodontist, is probably the main factor.”

In our sample, 13 patients out of 14 reported more than 1 partial loss of adhesion in the 12-year time span considered; in this group, 7 reported 4 or more losses, 2 reported 3 losses, and 4 reported 2 losses.

Therefore, while the large majority of patients did not experience failure of fixation, of those remaining that did have partial detachment, the majority had more than one. This fact suggests that these patients had unusual situations concerning detachment, e.g., having either precontacts or bruxism.

Precontacts could be signaled by early detachment occurrence and this seemed to be confirmed by the examination of clinical reports in 3 cases out of 14. However, more frequently, it seemed to be because of the occurrence of clinical signs of bruxism, such as facets or small fractures on teeth on disclusion pathways.

The more common visible clinical consequence of retention loss on single or multiple teeth was the ves-tibular or rotational dislocation of teeth, or frequently a combination of the two.

**Fig. 1:**
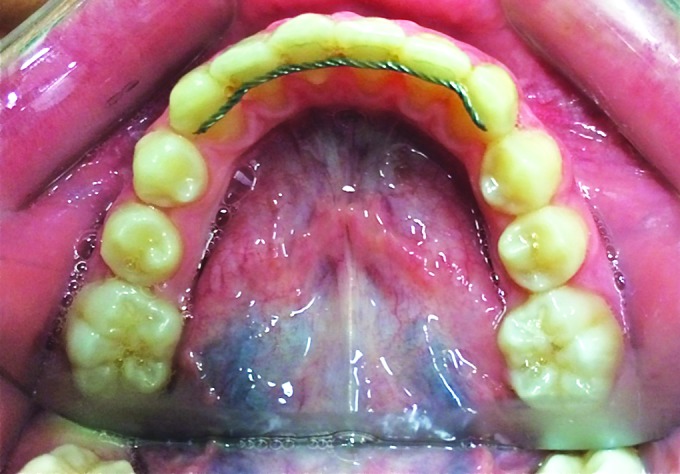
Loss of attachment with no dislocation

In the first place, evidence of teeth dislocation does not seem to be related to the time of loss of attachment but more frequently to the loss of a large part of the composite that fixes the retainer to the teeth. All teeth, which had detached but had maintained all composite material on the lingual, stayed in place and the loss of adhesion was revealed only by the hygienist or orthodontist (Fig. 1).

**Fig. 2:**
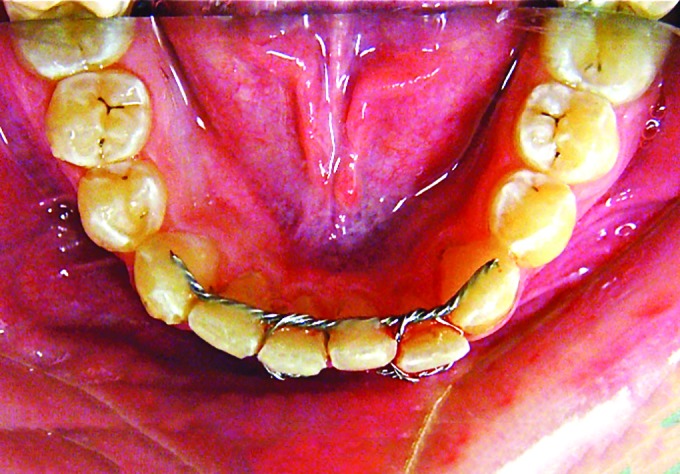
Tooth realignment with wire ligature

Nevertheless, dislocation was usually of little significance and never required intervention except in a single case where brackets had to be applied to realign teeth. All other teeth were realigned with the use of a circumferential wire ligature (Fig. 2).

On each occasion, separation between teeth and the retainer unit occurred in the enamel-composite and never in the wire-composite interface.

The observation that none of the patients examined in this study showed alterations of the gingival index in the area where the splint was applied, compared with other dentition areas of the same individual, confirms similar results and data obtained by authors, such as Heier, Gorelick, Dahl, Artun, Booth, and Renkema.^3,6-9,17^

In this study, the tendency for alteration in the gingival index to develop was more evident because the fixation of the splint was made on each tooth in the canine-incisor area; however, positioning of the wire around the cin-gulum and taking care not to overuse composite at the point of fixation seemed to solve the problem quite easily.

Nevertheless, the possibility of following the patients annually, thanks to the low-mobility occupations of people in our residential area, allowed clinicians to regularly check and solve problems in oral hygiene.

Of the 50 patients taken into consideration, only 2 showed carious lesions in the lower incisor area, and both of these were in the group of 46 that continued to wear the splint for the 12-year time span; only 18 others showed, in the same period, carious lesions in the posterior segment. In our opinion, these results are consistent with the hypothesis that the origin of carious lesions cannot be directly related to the presence of bonded retainers.

Notably, the two patients with lesions in the anterior region contemporarily had lesions in the posterior segments. Nevertheless, the two lesions were not in the lingual facets but in the vestibular one, and they were never in direct contact with wire or composite. As described earlier, the area of decalcification was observed in three patients. In all of these cases, the area of decalcification was in direct contact with the composite fixation of splints.

This evidence differs clearly with preceding notations; in any case, the lesions were present where the composite was not loose or displaced (even if it was disengaged). This fact suggests the possibility that it is retention of plaque and bacteria, even in patients with good oral hygiene habits, which influences the initial carious lesion. These patients did not develop complete lesions because annual checks enabled initial lesions to be found, thus preventing the possibility of plaque bacteria acting. This report stresses again the importance of regular control for the complete success of lingual retainers—both for perfect conservation of alignment and also for the prevention of enamel lesions.

In any case, all lesions were subclinical and were completely resolved during restoration of the retainers. Therefore, good compliance with this method of retention with fixed retention devices could be stated as the golden rule for long-term orthodontic treatment stabilization.

## CONCLUSION

All the available literature emphasizes the importance of using retainers in preventing relapses in orthodontic patients. Even if different strategies could be applied in retention, fixed retainers offer maximal assurance in maintaining teeth alignment in the lower anterior canine-incisor area.

Canine-only bonded retainers quite frequently resulted in the possibility of tooth movement, so single-tooth bonded fixation seems to offer some advantages in this direction.

The use of composite could facilitate bacteria and calculus retention, thus compromising periodontal and enamel status. The results of this study seem to confirm that lingual splints, if correctly applied and regularly maintained, do not increase the risk of periodontal damage or significantly influence the risk of caries.

This type of retention device seems to be accepted well by patients, even from a long-term perspective.

## CLINICAL SIGNIFICANCE

The present study demonstrates that the canine-to-canine bonded retainer is able to maintain good teeth alignment in the lower incisor area. Moreover, it seems not to be the primary cause of caries or periodontal problems in the frontal teeth, if the patient is able to maintain an appropriate oral hygiene. Therefore, fixed lingual splints represent an excellent device to avoid relapse after orthodontic treatment.
